# Genetics of strabismus

**DOI:** 10.3389/fopht.2023.1233866

**Published:** 2023-07-20

**Authors:** Mayra Martinez Sanchez, Mary C. Whitman

**Affiliations:** 1 Department of Ophthalmology, Boston Children’s Hospital, Boston, MA, United States; 2 Department of Ophthalmology, Harvard Medical School, Boston, MA, United States; 3 F.M. Kirby Neurobiology Center, Boston Children’s Hospital, Boston, MA, United States

**Keywords:** strabismus, genetics, esotropia, exotropia, copy number variant, linkage, GWAS, gene

## Abstract

Strabismus, or misalignment of the eyes, is the most common ocular disorder in the pediatric population, affecting approximately 2%–4% of children. Strabismus leads to the disruption of binocular vision, amblyopia, social and occupational discrimination, and decreased quality of life. Although it has been recognized since ancient times that strabismus runs in families, its inheritance patterns are complex, and its precise genetic mechanisms have not yet been defined. Family, population, and twin studies all support a role of genetics in the development of strabismus. There are multiple forms of strabismus, and it is not known if they have shared genetic mechanisms or are distinct genetic disorders, which complicates studies of strabismus. Studies assuming that strabismus is a Mendelian disorder have found areas of linkage and candidate genes in particular families, but no definitive causal genes. Genome-wide association studies searching for common variation that contributes to strabismus risk have identified two risk loci and three copy number variants in white populations. Causative genes have been identified in congenital cranial dysinnervation disorders, syndromes in which eye movement is limited or paralyzed. The causative genes lead to either improper differentiation of cranial motor neurons or abnormal axon guidance. This article reviews the evidence for a genetic contribution to strabismus and the recent advances that have been made in the genetics of comitant strabismus, the most common form of strabismus.

## 1 Introduction

Strabismus is the most common ocular disorder in children and can have lifelong consequences, including amblyopia, loss of binocular vision, decreased quality of life (for both children and parents), and social and occupational discrimination (1–7). Strabismus is classified by the alignment of the eyes relative to each other. When one eye is fixating, the other eye can deviate inwards (esotropia), outwards (exotropia), or vertically (hyper/hypotropia). Deviations can be the same in all positions of gaze (comitant strabismus) or vary based on eye position (incomitant strabismus). There are a group of rare syndromes (congenital cranial dysinnervation disorders, CCDDs) with incomitant, paralytic strabismus, which show typical Mendelian inheritance patterns, in which causative genes have been identified (8). Those identified genes influence cranial motor neuron development and affected patients and model organisms have abnormal development of the cranial nerves (9–13). This review focuses on the evidence for genetic contributions to the more common forms of comitant strabismus, in which both eyes are able to move fully. It has long been recognized that strabismus runs in families, and population, family, and twin studies all support a genetic contribution, but inheritance patterns are complex, and no definitive causative genes have been identified (14–20).

Genetic variants vary in both frequency (the prevalence in the population) and penetrance (the proportion of individuals with the genetic variant who have the disease). Mendelian disorders are usually caused by rare variants of high penetrance, whereas common diseases often have contributions of many common variants, each with relatively low penetrance (21). Different study techniques are used to identify these different types of variants, and both types of study have been performed in strabismus, which are detailed below.

## 2 Population differences

The prevalence of strabismus is approximately 2%–4% in the pediatric population (22–24), but the prevalence varies based on geographical regions and strabismus subtypes. A meta-analysis of 56 prevalence studies showed that the highest prevalence of esotropia and total strabismus was in the Americas and Europe, but that there was a higher prevalence of exotropia in Asia, especially China, and in Australia (25). A recent study in Hong Kong of ethnically Chinese children found a prevalence of strabismus of 3.1%, with an exotropia to esotropia ratio of 9.75 : 1 (26). Within the United States, the Multiethnic Pediatric Eye Disease study showed differences in the proportion of esotropia and exotropia between ethnic groups. Non-Hispanic white children had higher rates of esotropia than exotropia, whereas in African American, Asian, and Hispanic children exotropia was more common than esotropia (23, 27). The Baltimore Pediatric Eye Disease study, in contrast, reported that both white and black children had equal rates of esotropia and exotropia (24). Multiple other studies have reported that there is a higher prevalence of esotropia than exotropia in the white population (28–32), suggesting that population genetic differences contribute to the risk of esotropia in particular.

## 3 Risk factors

Strabismus has both genetic and environmental risk factors. In addition to family history, non-ocular strabismus risk factors include low birth weight, prematurity, maternal smoking during pregnancy, intrauterine drug exposure, neurologic diseases, and advanced maternal age (26, 33–38). Ocular risk factors include hyperopia (>2 D), and moderate anisometropia (26, 39), which likely have genetic risk factors. There is no significant difference in the incidence of strabismus between genders (40). Interestingly, several of these environmental risk factors have been associated with epigenetic changes, specifically changes in methylation, in the newborn population. Maternal smoking, birthweight, prematurity, and greater maternal age are all independently associated with altered methylation (41–44). Methylation patterns affect gene expression, suggesting that there is a possible mechanistic link between genetic and environmental risk factors for strabismus.

## 4 Twin and family studies

Hippocrates first noted that strabismus tends to run in families; he observed: “Children of parents having distorted eyes squint also for the most part” (45). Although genetic risk clearly plays a role in the development of strabismus (16), it is unknown whether esotropia, exotropia, and vertical deviations result from unique genetic risk factors, or if a common genetic risk underlies all forms of strabismus. In most families, all affected individuals have the same general type of strabismus (esotropia *vs*. exotropia), but there are examples of families that are discordant (46–49). It is clear that there is not one single gene that accounts for all strabismus cases.

Twin studies support there being a strong genetic contribution, particularly for esodeviations (20). Monozygotic twins have concordance rates for any strabismus of 54%–82%, while dizygotic twins have concordance rates of 14%–47% (19, 50). Several family studies have shown a high prevalence of strabismus among family members of a proband with strabismus (15, 51–53). Combining 12 previous family studies, Paul and Hardage (16) reported that 30.6% of strabismus patients had a close relative with strabismus. A more recent study reported that 18.7% of strabismus cases had at least one affected first-degree relative (54). The relative risk for first-degree relatives of an affected proband is estimated to be between 3 and 5 (14, 16–19). The heritability factor remains significant following correction for the environmental risk factors identified above (17).

## 5 Linkage analysis in large strabismus families—is strabismus a Mendelian disorder?

In large strabismus pedigrees in which strabismus is inherited in apparently Mendelian patterns, several studies have used linkage analysis to identify potential loci associated with strabismus (Table 1). A locus on 7p22.1 (STBM1) was mapped in one large family, under a recessive model, although inheritance appeared to be dominant (18). This locus was replicated in another family under a dominant model (55). By combining Japanese families, linkage was shown at 4q28.22 in a dominant model and 7q31.2 in a recessive model (56). *MGST2* and *WNT2* have been proposed as candidate genes at these loci, but specific variants have not been reported (61). In one large family, with variable strabismus phenotypes between family members, linkage was shown at 14q12; the top candidate variant is a non-coding 4 bp deletion near *FOXG1* (59). There have also been two studies in large consanguineous families in which some individuals had infantile esotropia and others had Duane retraction syndrome. The first study identified a recessive 6MB band on chromosome 16p13.12-p12.3 (57); the other study identified two potential loci (3p26.3–26.2 and 6q24.2–25.1) in a family with three siblings with esotropia and four with Duane retraction syndrome (58). In one large Chinese family, linkage was identified at 2q22.3–2q32.1, and exome sequencing identified a rare heterozygous variant in *LRP2* within the area of linkage. An additional rare variant in the same gene was identified in an unrelated family (60). Exome analysis of another Chinese family, in which the proband, both parents, and a maternal grandfather had exotropia, identified several candidate variants, but no functional evidence was presented for any of the variants (62). Another study reported an exome analysis of 18 strabismus families. In five families, a potential risk variant—defined as a rare, predicted deleterious variant that segregated with disease—was identified. The genes with potential risk variants are *FAT3*, *KCNH2*, *CELSR1*, and *TTYH1* (63). Since each of these studies has identified different potential risk variants in different families, it is clear that there is significant genetic heterogeneity in strabismus.

## 6 GWAS of strabismus—is strabismus a common disorder?

Diseases that are common in the population often have multiple genes that contribute, with each individual variant having a small effect size. In search for common variations contributing to the risk of strabismus, two genome-wide association studies (with different inclusion criteria) have each reported one risk allele (64, 65). A GWAS study of non-accommodative esotropia, including 826 patients of European ancestry from the USA in the discovery cohort and 689 patients from Australia and the United Kingdom in the replication cohort, identified one risk allele, a functional single nucleotide polymorphism (SNP) in an intron of the WRB gene on chromosome 21 at rs2244352, which affects the expression of WRB and neighboring genes (64). The area is imprinted, and the risk allele is preferentially inherited paternally (64). A second GWAS, including 1,345 individuals with self-reported strabismus in the UK Biobank, identified a locus on chromosome 17q25, with lead SNP within *TSPAN10* (65). The 17q25 locus extends across the *NPLOC4*–*TSPAN10*–*PDE6G* gene cluster, which has been associated through GWAS with several other eye conditions, including macular thickness (66), astigmatism (67), retinal microvascular size (68), and myopia (69). Phenotyping in the UK Biobank was based on self-reporting by participants aged between 37 and 73. Specifically, participants reported whether they wore glasses and, if they did, were asked to select the reason(s). Participants who chose “squint or turn in an eye since childhood (called strabismus)” were classified as having strabismus. This has the potential to select for accommodative esotropia (and associated hyperopia) or to misclassify participants with strabismus as controls if they do not wear glasses for the condition. The strabismus group was more hyperopic than the control group, but the association between strabismus and the 17q25 locus was independent of refractive error. The association with this locus was replicated in a cohort of 7-year-olds examined by an orthoptist and determined to have strabismus. In that population of 5,200 children (of whom 145 had strabismus), the lead variant was associated with any strabismus and exotropia, but not with esotropia independently. The authors note, however, that some of the exotropic children might have been exotropic following surgery for esotropia and that the number of exotropic children was quite small (only 28) (65). The association of each of these alleles with strabismus was replicated in the FinnGenn cohort, in which patients were phenotyped based on electronic health record data. In the Finnish cohort, which comprised 3,515 cases of all subtypes of strabismus (including convergent, divergent, paralytic, and vertical strabismus, accommodative esotropia, intermittent heterotropia, and others) and 173,384 controls, the WRB polymorphism was associated with “any strabismus” and “divergent strabismus”, and the TSPAN10 polymorphism was associated with “any strabismus”, “convergent strabismus”, and “divergent strabismus” (70). Overall, the data indicate that these two polymorphisms contribute significantly to the population-attributable risk of strabismus, but more GWAS studies, with larger sample sizes and consistent phenotyping, are needed (71).

## 7 Copy number variants (CNVs) in strabismus

Additional sources of genetic variation in the population are copy number variants (CNVs) and structural variants. CNVs are duplications or deletions of large regions of DNA, whereas structural variants are rearrangements of the DNA, such as inversions, insertion of sequence from one chromosome to another, or translocation between chromosomes. CNVs represent an important source of genetic variation and are also a force that shapes genome evolution (72, 73). Many common CNVs represent benign polymorphisms, but rare CNVs at specific loci contribute to disease. CNVs contribute to the genetic risk for several neuropsychiatric disorders with complex inheritance (74), including intellectual disability (75), autism spectrum disorder (76, 77), major depressive disorder (78), attention-deficit hyperactivity disorder (79), and Tourette’s syndrome (80). In strabismus, one study has reported both a larger number of CNVs in esotropia samples and three specific duplications that significantly increase the risk of esotropia (81). They compared 1,614 white individuals with isolated esotropia to 3,992 ethnically matched control individuals, all genotyped on Illumina Omni SNP arrays, and used two hidden Markov model (hmm) CNV-calling algorithms, PennCNV (82) and QuantiSNP (83), to identify CNVs. Three rare, recurrent duplications were significantly (p <1 × 10^−6^) more common in esotropia patients. A 23 kb duplication at 4p15.2 includes exon 1 of the uncharacterized lncRNA *LOC101929161* (also known as lnc-SEL1L3–2) and shows conservation with monkeys, but not other animals. The duplication on chromosome 2p11.2 spans the lncRNA *CYTOR* and microRNA *miR4435*, contains several putative regulatory regions, and has areas with conservation among mammals but not other vertebrates. The duplication on chromosome 10q11.22 spans two lncRNAs: *LINC00842* and *LOC105378577*; three protein-coding genes: *ANTXRL* (anthrax toxin receptor-like), *ANXA8L1* (annexin A8 like 1), and *NPY4R* (neuropeptide Y receptor 4); and three transcribed pseudogenes: *ANTXRLP1*, *FAM25BP*, and *HNRNPA1P33.* Each duplication substantially increased esotropia risk [OR 11.1 (95% CI 4.6–25.2), OR 14.16 (95% CI 5.4–38.1), and OR 8.96 (95% CI 5.4–14.9), respectively]. Further study to identify the mechanisms by which these duplications contribute to strabismus is ongoing.

There are many reports of copy number variants associated with neurodevelopmental disorders that include strabismus (see section 9). In neurotypical patients, two cases who presented with Kallmann syndrome, X-linked ichthyosis, and strabismus, who have partially overlapping deletions on the X chromosome, have been reported. The deletion of *ANOS1* and *STS* explains the Kallmann syndrome and X-linked ichthyosis, respectively. The other four genes deleted in both patients are *NLGN4X, VCX-A, PUDP*, and *PNPLA4*. Neither patient had an intellectual disability or developmental delay (84).

## 8 CCDDs

To date, the only causal genes for strabismus have been found in CCDDs. Patients with ocular CCDDs have paralysis of one more extraocular muscles, due to abnormalities of neuronal differentiation or axon guidance of the corresponding cranial motor nerve (85). Congenital fibrosis of the extraocular muscles (CFEOM) affects cranial nerves 3 and 4 and is caused by autosomal dominant missense variants in *KIF21A*, *TUBB3*, *TUBB2B* or *TUBA1A* or autosomal recessive variants in *PHOX2A* (9, 10, 12, 86, 87). Duane retraction syndrome affects cranial nerve 6, leading to limited abduction of one or both eyes. Although most cases do not have an identifiable genetic cause, autosomal dominant variants in *CHN1* or *MAFB* can cause isolated Duane syndrome (11, 88), and variants in *SALL4* cause Duane-radial ray syndrome (Duane syndrome plus abnormalities of forearm development) (89). Duane syndrome is also a feature of Athabascan brain dysgenesis syndrome (ABDS)/Bosley–Salih–Alorainy syndrome (BSAS), caused by autosomal recessive variants in *HOXA1* (90, 91). Horizontal gaze palsy with progressive scoliosis (HGPPS) involves an inability to abduct or adduct the eyes, and results from autosomal recessive variants in *ROBO3* (92). *HOXB1* variants result in a syndrome that includes facial paresis and esotropia with full eye movements (93). It is clear, however, that comitant strabismus results from different mechanisms than the CCDDs, since patients with comitant strabismus have full motility and normal cranial motor nerve function and appearance on MRI.

## 9 Genetic syndromes including strabismus

Although comitant strabismus is most often an isolated ocular disorder, it is also a common feature of many genetic syndromes and neurodevelopmental disorders (16, 94). The incidence of strabismus in individuals with Down syndrome is 33% (95); strabismus has also been reported with many other chromosomal disorders associated with intellectual disability, including partial trisomy 7q (96, 97), partial trisomy 10p (98), and duplication 13q (99). Individuals with intellectual disability of any cause are significantly more likely to have strabismus than the general population; the odds ratio is 5.46 (100). The incidence of strabismus in individuals with autism spectrum disorder is also increased (8%–22%) (101–104). Ye et al. (105) compiled a list of 233 genes potentially involved in strabismus that was based on strabismus being a feature of their associated genetic syndrome. Analysis of these potential genes reveals their preferential expression in the cerebellum, amygdala, and posteroventral parietal cortex, specifically at the fetal and early developmental stages.

Strabismus is also common in ocular disorders that lead to poor vision, including retinal dystrophies, congenital cataract, optic nerve hypoplasia, and albinism (106–109). Poor vision contributes to the development of strabismus in these disorders, but it is also possible that the genetic changes that lead to retinal, optic nerve, or lens maldevelopment or dysfunction affect the oculomotor system.

## 10 Conclusion

Although strabismus has a clear heritability, its genetic mechanisms are complex and heterogeneous. Several linked loci and candidate genes have been identified in individual families, but, thus far, exome sequencing has not revealed definitive causative variants present in significant proportions of strabismus patients. Across the population, two loci have been identified by GWAS as increasing the risk of strabismus, each with a fairly small effect size. Three CNVs increase the risk of strabismus; they have large ORs but are rare. Many neurodevelopmental disorders include strabismus; one hypothesis is that isolated strabismus may result from altered regulation of genes whose loss of function causes more severe neurologic dysfunction. Altered genetic regulation may be a common mechanism that links all the risk factors for strabismus. CNVs may alter gene regulation by disrupting the proximity of enhancers and silencers to promoters, the SNPs identified by GWAS affect the regulation of nearby genes, and the environmental risk factors affect epigenetic methylation, which in turn affects gene regulation. Further study and improved methods of identifying variants in regulatory regions are needed to fully understand the genetic causes of strabismus.

## Figures and Tables

**TABLE 1:** Common strabismus loci identified by linkage analysis.

Locus	Reported LOD	Potential gene	Phenotype	Model	Ethnicity	Reference
7p22.1	4.51		ET	Recessive	European	(18)
7p22.1	3.21		ET	Dominant	European	(55)
7q31.2	4.4	*WNT2*	ET + XT	Recessive	Japanese	(56)
4q28.3	3.62	*MGST2*	ET + XT	Dominant	Japanese	(56)
16p13.12-p12.3	2.5		ET + Duane + double elevator palsy	Recessive	Saudi Arabian	(57)
3p26.3–26.2	3.18		ET + Duane	Recessive	Saudi Arabian	(58)
6q24.2–25.1	3.25		ET + Duane	Recessive	Saudi Arabian	(58)
14q12	4.69	*FOXG1*	Mixed	Dominant	European	(59)
2q22.3–2q32.1	3.54	*LRP2*	XT	Dominant	Chinese	(60)
